# Quasi-Solid-State Electrochromic Cells with Energy Storage Properties Made with Inkjet Printing

**DOI:** 10.3390/ma13143241

**Published:** 2020-07-21

**Authors:** Krystallia Theodosiou, Panagiotis Giannopoulos, Tilemachos Georgakopoulos, Elias Stathatos

**Affiliations:** 1 BRITE Solar Technologies, Patras Science Park, Stadiou Str., Platani, 26504 Patras, Greece; ktheodosiou@britesolar.com (K.T.); pgiannopoulos@britesolar.com (P.G.); tgeorga@britesolar.com (T.G.); 2 Nanotechnology and Advanced Materials Laboratory (NAML), Electrical and Computer Engineering, University of the Peloponnese, 26334 Patras, Greece

**Keywords:** electrochromic cells, energy storage, cerium-modified Titanium dioxide, quasi-solid-state electrolyte

## Abstract

In common commercially available electrochromic glass panes, the active materials such as WO_3_ and NiO_x_ films are typically deposited by either physical vapor or sputtering under vacuum. In the present studies, we report on the inkjet printing method to deposit both electrochromic and ion storage electrode layers under ambient conditions. An ion storage layer based on cerium modified TiO_2_ and electrochromic nanocrystalline WO_3_ were both prepared under the wet method and deposited as inks on conductive substrates. Both compounds possess porous morphology facilitating high ion diffusion during electrochemical processes. In particular, the ion storage layer was evaluated in terms of porosity, charge capacity and ion diffusion coefficient. A scaled up 90 cm^2^ electrochromic device with quasi-solid-state electrolyte was made with the aforementioned materials and evaluated in terms of optical modulation in the visible region, cyclic voltammetry and color efficiency. High contrast between 13.2% and 71.6% for tinted and bleached states measured at 550 nm was monitored under low bias at +2.5 volt and −0.3 volts respectively. Moreover, the calculated energy density equal to 1.95 × 10^−3^ mWh cm^−2^ and the high areal capacitance of 156.19 mF cm^−2^ of the device could combine the electrochromic behavior of the cell with energy storage capability so as to be a promising candidate for future applications into smart buildings.

## 1. Introduction

The high need for green technology and reduced energy consumption over the last decades has led to the development of various chromogenic materials. Chromogenic materials have the ability to change their optical properties as a response to an external stimulus. Typical examples are photochromic, thermochromic and electrochromic materials [1,2,3]. The materials that are able to change their optical properties, when an electric potential is applied are called electrochromic (EC) and probably have been most studied among the rest of the chromogenic materials. This optical change is reversible after suitable bias and is accompanied by injection and extraction of electrons and small ions (such as H^+^ and Li^+^) [4,5]. An ideal EC device would provide high optical contrast, fast switching time between colored and clear states, long cycle life and low manufacturing cost [6]. Due to the high need for significant energy saving, the current electrochromic research is aiming to develop electrochromic windows, usually called "smart" windows. A smart window is applicable across all building types and can be used in order to achieve improved energy efficiency, through the control of the transmittance of sunlight and solar heat into a building [7,8,9].

**Figure 1:**
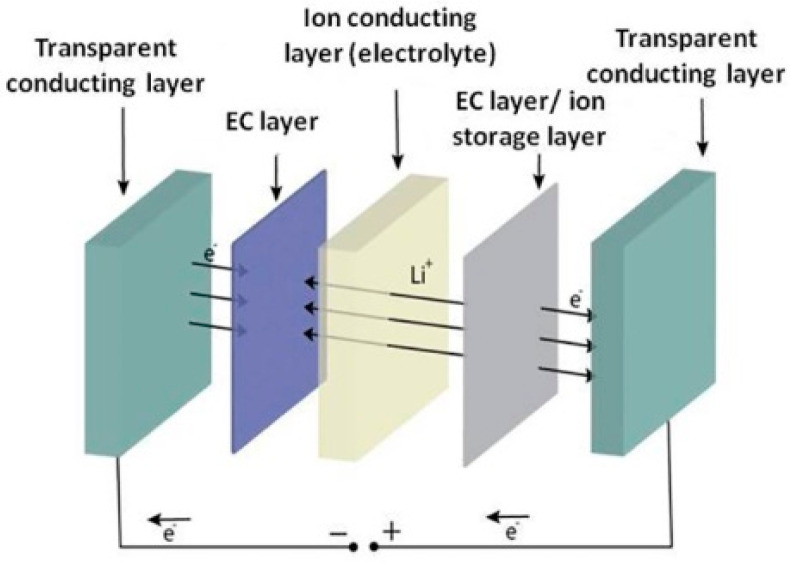
Typical structure of an electrochromic (EC) device.

A typical EC device consists of five layers, as presented in Figure 1: two transparent conducting layers (typically conductive glass substrates, SnO_2_:F (FTO)), the EC layer, an ion storage layer and an ion conductor among them, typically referred to as electrolyte in liquid systems [10]. The EC layer may consist of a "cathodic" EC material (materials that can be colored under cation insertion), or an "anodic" EC material (coloration under anion insertion). However, an EC device could also participate with EC layers of both types, one of them consisting of cathodic EC material and the other consisting of anodic EC material. Both layers have to color simultaneously under a specific external bias voltage, in order to achieve the maximum device coloration. A great number of materials, such as organic molecules, conducting polymers and transition metals, have been used for the development of EC devices. Tungsten(VI) trioxide (WO_3_) is the most widely studied electrochromic material. WO_3_ can be colored under cation or proton insertion (cathodic EC material).

Titanium dioxide (TiO_2_), Niobium pentoxide (Nb_2_O_5_) and Molybdenum trioxide (MoO_3_) have also been used as cathodic EC materials. The films based on the above materials have the ability to change their color from transparent or pale yellowish to blue or brown, due to electrochemical reduction. Among all electrochromic materials, WO_3_ is considered to have the best overall performance, due to its high coloration efficiency, good cycling life and fast switching time. On the other hand, the majority of the rest of the widely studied cathodic EC materials have shown either low coloration efficiency, or poor durability over electrochemical cycling [6,11,12,13,14]. The electrochemical reduction that leads to the coloration of WO_3_ can be written as:

(1) $$\underset{\mathrm{transparent}}{WO_{3}}+x\left(A^{+}+e^{-}\right)\to A_{x}W^{Vl}{}_{\underset{\mathrm{blue}}{\left(1-x\right)}}W_{x}^{V}O_{3}$$

which A^+^ refers to H^+^, Li^+^, Na^+^;

Anodic EC materials can be colored by electrochemical oxidation and are usually employed as counter electrodes (CE). The most popular anodic EC material is nickel oxide (NiO), whereas iridium dioxide (IrO_2_), vanadium pentoxide (V_2_O_5_), and organic-, metallo-organic or polymeric complexes have also been used [15,16,17,18,19,20,21]. The electrochemical reaction of NiO coloration in alkaline aqueous solution can be described as:

(2) $$\underset{\mathrm{transparent}}{NiO}+OH^{-}\to \underset{\mathrm{brown}}{NiOOH}\;+e^{-}$$

In a different approach, when a second EC material is not used as it is difficult to achieve a simultaneous operation of both electrochromic materials by the same electrolyte, an optically passive layer can be used as the counter electrode. Films based on cerium dioxide (CeO_2_), tin dioxide (SnO_2_) or zirconium dioxide (ZrO_2_) have been widely used [22,23,24,25]. In this approach, the coloration of the EC device is due to the coloration of only one EC layer. The optically passive layer acts as an ion-storage layer and presents high and stable optical transmittance in both oxidized and reduced states. In a slightly modified approach, one electrode can act simultaneously as electrochromic and energy-storage layer [26].

There are several methods that have been tested for the preparation of EC layers or ion-storage layers [27,28]. Most of them are solution-based, such as spray pyrolysis [29,30], the sol-gel method [31,32] and electrodeposition [33,34]. Among these, the sol-gel process is low-cost and can be easily used for the preparation of large area films, through various techniques. One of the most promising techniques, employing sol-gel prepared solutions, is inkjet printing. The inkjet printing process has the advantage of precise, high speed and contact-less deposition in single- or multi-layer constructions at a very affordable price [35,36]. Additionally, through inkjet printing the material wastes are limited, the process is quite simple and applicable to a variety of substrates and high resolution can be achieved [37,38,39]. On the other hand, it is very important to control the ink properties (viscosity, surface tension and evaporation rate) and printing settings, in order to achieve a very homogenous film [40]. In consequence, even if a great number of works over recent years have focused on inkjet printing technology, there is no standard protocol for the preparation of suitable inks for inkjet printing and various obstacles have been observed (unstable inks, inhomogeneous films, nozzle clogging and so on) [41].

Finally, the selection of an appropriate ion conductor (electrolyte) is also a critical parameter for the electrochemical process. The electrolyte must be chemically stable under electrochemical cycling, with as fast a response time as possible. Common organic solvents are usually not used, due to their toxicity, flammability and high volatility [42,43]. Therefore, gel electrolytes based on polymers or ionic liquids (ILs) are much more desirable. Gel electrolytes have been used in various applications such as dye-sensitized solar cells, batteries and supercapacitors, whereas they present higher ionic conductivity than solid electrolytes [44,45,46,47,48,49].

In the present work, we report on the preparation of a quasi-solid-state EC device based on a WO_3_ EC layer and an ion-storage layer based on cerium-modified TiO_2_. The final solutions were prepared by a very simple procedure, without steps such as centrifugation, filtration or other techniques that usually are necessary. The above electrodes were prepared using the inkjet-printing method by suitable deposition of inks composed of the aforementioned materials on FTO glass, while their morphological and electrochemical properties have been investigated. The electrolyte used was based on a nanocomposite organic-inorganic gel, prepared using the sol-gel procedure. Through this route, the electrolyte provides good ionic conductivity and simultaneously works as a gluing material holding the two electrodes together. The above device presents excellent electrochemical stability and optical reversibility between clear and colored states, and has supercapacitance properties too. The specific approach is very promising and can be used in large-scale production for the preparation of smart windows. Our goal was the preparation of samples that can be easily produced and used in large scale applications, so great attention was paid to the detail, in order to achieve a fully homogeneous sample, free of even small irregularities that are usually observed in most research works.

## 2. Materials and Methods 

### 2.1. Chemical Materials and Characterization Techniques

Commercial titanium (IV) isopropoxide (TTIP, 97%, Sigma-Aldrich, St. Louis, MO, USA), Triton X-100 (Sigma-Aldrich, St. Louis, MO, USA), glacial acetic acid (AcOH, 99–100%, Sigma-Aldrich, St. Louis, MO, USA), ethanol (absolute, 99.5% Sigma-Aldrich, St. Louis, MO, USA) and cerium(III) nitrate hexahydrate (90%, Sigma-Aldrich, St. Louis, MO, USA), were used to make Ce modified-TiO_2_ precursor sols. Tungsten powder (99.9%, Sigma-Aldrich, St. Louis, MO, USA) and hydrogen peroxide solution (30% in water, Sigma-Aldrich, St. Louis, MO, USA) were used to prepare the WO_3_ precursor sol. All solvents were purchased from Sigma-Aldrich and they were used as received. SnO_2_:F transparent conductive electrodes (FTO, TEC^TM^ A8) with 8 Ω/sq were purchased from Pilkington NSG Group (Tokyo, Japan).

O,O’-Bis(2-aminopropyl) polypropylene glycol-block-polyethylene glycol-block-polypropylene glycol (Jeffamine® ED-600, Mr~600) and 3-isocyanatopropyltriethoxysilane (ICS; molar ratio ICS/diamine = 2) were used for the synthesis of hybrid organic/inorganic material abbreviated as ED-600-ICS which were put in a vessel to react (acylation reaction), producing urea connecting groups between the polymer units and the inorganic part. The as-prepared hybrid organic/inorganic material ED-600-ICS is necessary for the quasi-solid-state electrolyte according to the procedure described in our previous publications [50,51].

Electrochemical measurements were carried out in a three-electrode cell with the use of an Autolab PGSTAT 302Ν electrochemical analyzer (Metrohm AG, Herisau, Switzerland). All experiments were carried out in freshly prepared 1.0 mol L^−1^ LiClO_4_ in PC as the electrolyte. The working electrode was either the WO_3_ film or the cerium-modified TiO_2_ film deposited on FTO coated glass, the reference electrode was Ag/AgCl and the counter electrode was a platinum foil. In particular, for the double-junction electrode Ag/AgCl, the reference electrolyte was LiCl 2mol/L in ethanol and the bridge electrolyte was LiClO_4_ 1 mol/L in propylene carbonate (PC). The ultraviolet-visible/NIR absorption diffuse reflectance spectra of the electrochromic films and devices were obtained at ambient conditions in a range of 300 to 2500 nm using a Jasco V-770 spectrophotometer (Jasco, Easton, PA, USA) equipped with a 60 mm integrating sphere embedding a PbS detector (ISN-923). Nanostructure morphology of the films was studied by field-emission electron microscopy (FE-SEM, Zeiss SUPRA 35VP) and TEM microscopy, JEOL (JEM-2100, Yamaguchi, Tokyo). As the porosity of the ion storage layer in electrochromic cells is very important the stuctural properties of the Ce modified-TiO_2_ were also evaluated with porosimetry. Nitrogen adsorption/desorption curves for these samples were measured with a Micromeritrics Tristat 3000 and the surface area and pore size distribution were derived by differentiating them according to the BET method. Finally, the inkjet printing technique was performed using the Fujifilm Dimatix printer (DMP2850); the printheads that were employed for film deposition of all materials consisted of sixteen nozzles depositing 10 pl drop volumes.

### 2.2. Development of Electrochromic Materials and Films

#### 2.2.1. Tungsten Trioxide Thin Layers

The preparation of tungsten trioxide (WO_3_) films was carried out by an inkjet printing technique on FTO glass substrates. The first step involved a highly exothermic reaction between 5 g of W powder and 20 mL of hygrogen peroxide (H_2_O_2_), which led to the formation of peroxotungsten acid (PTA) [33]. In the above solution, a mixture of Triton X-100 and 2-Propanol with an optimized molar ratio 1/70 = Triton X-100/2-Propanol was then added. The final ink contained 28 mmol of tungsten per 60 mL of sol. Triton X-100, a nonionic long chain surfactant, was selected as a pore directing agent in the solution. Compared to other commonly used toxic and ionic templating agents, Triton X-100 is relatively inexpensive, non-toxic, and easily removable. Such amphiphilic molecules exhibit ordered mesophase and the ability to adjust large inorganic clusters in aqueous condition [52,53]. The final tungsten ink had surface tension equal to 30 dynes/cm, viscosity equal to 11 cPs, density equal to 1.1 g/ml and was filtered through a 0.2 μm PTFE filter before being used in the printing procedure. Small and spherical drops were achieved, while a 20 μm drop spacing was employed. The reliability and repeatability of the film was excellent during printing. The film was deposited on SnO_2_:F (FTO) coated glass 3.2 mm thick. Before deposition, the substrates were cleaned with soft detergent, rinsed with deionized water and calcined at 500 °C for 10 min in order to remove moisture and any organic residues. The as-prepared WO_3_ films were uniform and optically transparent.

#### 2.2.2. Cerium Modified-TiO_2_ Thin Films as Ion Storage Layers

A suitable amount of Triton X-100 was homogeneously dissolved in ethanol (EtOH). Before adding titanium(IV) isopropoxide precursor (TTIP), AcOH was added into the solution. Then, the TiO_2_ precursor TTIP was added during vigorous stirring. The molar ratio of the materials was optimized at Triton X-100:EtOH:AcOH:TTIP = 1:69:6:1. Cerium(III) nitrate was finally added to the previous solution in various quantities before we concluded determination of the optimal ratio. We finally kept 10% weight ratio of cerium nitrate in the TTIP solution as the optimized concentration for our experiments because the electrochromic properties of our devices were also optimized. The final ink was printed using the same parameters as for the tungsten ink since the physical properties of the two inks were comparable. After several minutes, the dispersion was ready to be used on glass slides. Films prepared on FTO glass were also prepared with the inkjet printing process as in the case of WO_3_.The as-prepared films were also uniform and optically transparent as in the case of WO_3_.

#### 2.2.3. Synthesis of Quasi-Solid-State Electrolyte and Completion of the EC Device

1.2 grams of sulfolane (>99.5%, Sigma-Aldrich, St. Louis, MO, USA) were mixed with 0.29 grams of ED-600-ICS organic-inorganic hybrid substance followed by an addition of 0.092 grams of acetic acid (>99.85%, Alfa Aesar, Haverell, MA, USA), and finally of 0.082 grams of anhydrous LiClO_4_ (>99.5%, Sigma-Aldrich, St. Louis, MO, USA). The quasi-solid-state electrolyte was prepared just before the completion of the EC assembly to avoid any gelation process to start before finalizing the device. After preparing the aforementioned films by inkjet printing, we used them as electrodes in order to fabricate electrochromic cells of the following configuration: Glass/FTO/WO_3_/electrolyte/Ce-modified TiO_2_/FTO/Glass. Electrochromic cells with typical dimensions of 9 cm × 10 cm were prepared as follows: The anode FTO/Ce-modified TiO_2_ and the cathode FTO/WO_3_ were placed opposite to each other with their conductive sides slightly removed so as to maintain the appropriate space for electrical contacts. The space between the two electrodes was filled with the electrolyte. The liquid form of the electrolyte was inserted through a hole that was drilled into one of the two glasses, while the space was fixed in place to a distance of 50 micrometers by the application of thermoplastic material (DuPont Surlyn®) around the glass. After 30 min the electrolyte turns to a gel, due to the esterification reaction of the alkoxy groups at both edges of the hybrid material ED-600-ICS.

## 3. Results and Discussion

### 3.1. Microstructure Properties of Both Electrodes

#### 3.1.1. WO_3_ Films

**Figure 2:**
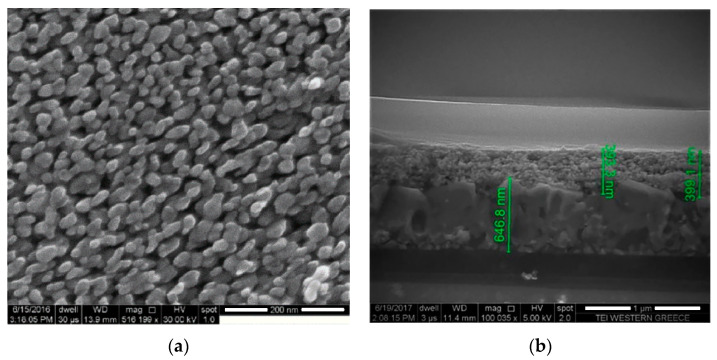
(**a**) Top view field-emission electron microscopy (FESEM) image and (**b**) cross section image of WO_3_ films.

The analysis of the structure and morphology of the WO_3_ films were carried out using scanning electron microscopy (SEM). Figure 2a shows the SEM image of the surface of the WO_3_ film printed on FTO coated glass. The film seems to be homogeneous over the entire surface area. Furthermore, it is obvious that particles formed with sizes in the range of 20–40 nm. It is also obvious from the image that WO_3_ particles are not agglomerated in high grade, creating a porous structure within the film. Figure 2b illustrates the cross section image of the as-prepared film. It can be seen that the film was successfully deposited on the substrate and the thickness of the layer was between 390 to 400 nm. In addition, it is clear that the electrochromic layer is not compact in contrast to the FTO layer (647 nm) on the bottom, which facilitates the electrolytes’ pouring into the pores of WO_3_ film.

#### 3.1.2. Ce-Modified TiO_2_ Films

**Figure 3:**
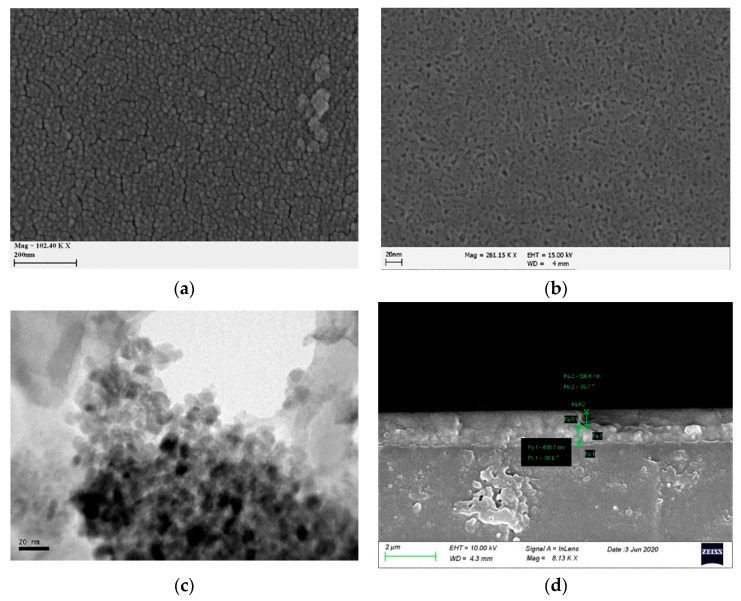
Top-view of (**a**) TiO_2_, (**b**) Ce- modified TiO_2_ film, (**c**) TEM image of particles scratched from a Ce- modified TiO_2_ film and (**d**) cross section view of a Ce- modified TiO_2_ film (from [55] with copyright permission from Elsevier).

The morphology of the Ce-modified TiO_2_ films employed as ion storage layers in EC devices is presented in Figure 3. Moreover, we also present pristine TiO_2_ films for comparison with those modified with cerium. The films proved to be in both cases very homogenous while in the case of Ce-modified TiO_2_ the particles were even smaller. This was verified by the TEM image shown in the same figure. The average size of TiO_2_ particles was 10–12 nm (Figure 3a) while in the case of 10% Ce-TiO_2_ the size was even smaller (7–8 nm) (Figure 3b). The decrease of particle size can be attributed to the specific interaction of Ce ions at the interface of titanium dioxide particles, which prevents TiO_2_ anatase particles from adhering together and inhibits the growth of crystal grains [54]. We examined the XRD spectrum of the composite film containing Ce ions (10% w/w) where a new peak at about 2θ = 30.8° (Figure S1) was also observed among the characteristic peaks of anatase [JCPDS ICDD card No. 21-1272]. It most probably means that a new crystal phase corresponding to cerium titanate Ce_x_T_(1-x)_O_2_ was formed. The intensity of this peak increased along with the concentration of Ce ions in the starting sol. This peak cannot be attributed to common cerium oxides (CeO_2_, Ce_2_O_3_, CeO, etc.). However, a more detailed examination of the spectrum may indicate the existence of brookite in XRD patterns, which is evidenced from the presence of the (211) peak at 2θ = 30.68^ο^ (JCPDS ICDD cards No. 76-1937). However, the cerium atoms are undoubtedly present in the films and for that reason we prefer the term cerium modified TiO_2_ films. As the particles’ dimensions are very small in the case of Ce-modified TiO_2_, a TEM image was selected to fully characterize and certify the accurate size in this case (Figure 3c). Our initial predictions were proved correct, as it was found that the size of the particles was around 8 nm. The thickness of the Ce-modified TiO_2_ films was around 590 nm according to the cross section image of (Figure 3d).

### 3.2. BET Surface Areas for Ce-TiO_2_ and Pristine TiO_2_ Films

**Figure 4:**
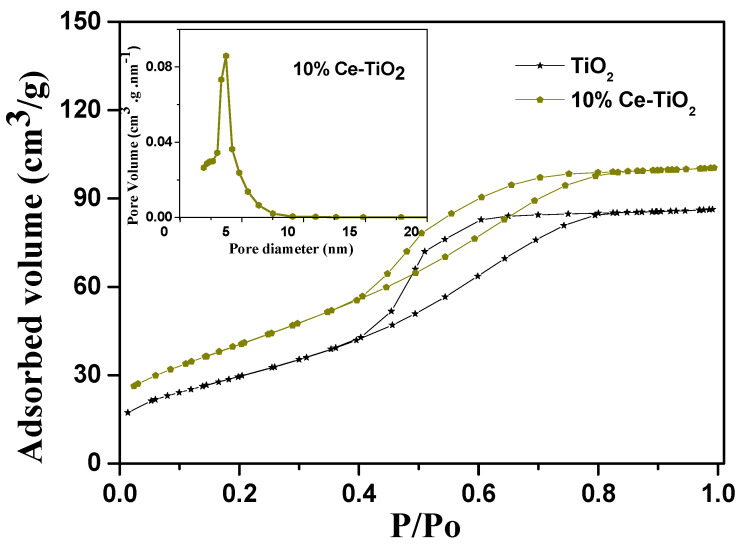
Sorption–desorption curves for pristine TiO_2_ and cerium-modified TiO_2_ films and pore size distribution (inset) of 10% cerium-modified TiO_2_ films (adapted from [55] with copyright permission from Elsevier).

Porosity of the ion storage layer is very important to the operation of electrochromic devices, therefore we performed measurements to have an estimation of this parameter. Nitrogen sorption–desorption isotherms and the corresponding pore size distribution curve are shown in Figure 4. The isotherms for the samples of 10% Ce-TiO_2_ and pristine TiO_2_ used as a comparison show that the sorption starts at P/P_0_ ≈ 0.5–0.8; that is due to the capillary condensation in the mesoporous structure while the pore size distribution is narrow and centers at around 7 nm. The BET specific surface area (SSE) for Ce-modified samples is relatively high and was calculated at 151 m^2^/g compared to 121 m^2^/g for pristine TiO_2_. The total pore volume (V_p_) was measured at 0.220 cm^3^/g for cerium modified TiO_2_ films, while it was found to be 0.133 cm^3^/g for pristine TiO_2_ films. From data presented for both samples it is obvious that the presence of cerium leads to higher values for SSE, which is a promising result for the as-prepared cerium-modified TiO_2_ films to be employed as ion storage films in electrochromic devices. The porosity of the films was also calculated to be 34% and 43% for pristine TiO_2_ and cerium-modified TiO_2_ samples, respectively.

### 3.3. Electrochemical Characterization of Electrochromic and Ion Storage Film.

The electrochemical properties of electrochromic and ion storage films, developed in this work, were investigated using the cyclic voltammetry technique. Electrochemical measurements were carried out in a three-electrode cell with the use of an Autolab PGSTAT 302Ν electrochemical analyzer connected to a personal computer running NOVA software (Version 1.8, Metrohm AG, Herisau, Switzerland) in order to collect and analyze the experimental data. The working electrode was either the WO_3_ film or the cerium-modified TiO_2_ film deposited on FTO coated glass, the reference electrode was Ag/AgCl and the counter electrode was a platinum foil. A solution of 1M LiClO_4_ in propylene carbonate served as the electrolyte. Cyclic voltammetry measurements for assessment of the WO_3_ film were recorded with a linear potential sweep between −0.8V and +1.2V at various scan rates. It was observed that during the cathodic potential scan the film changed from colorless to deep blue, which indicated WO_3_ reduction, followed by intercalation of lithium ions. On the other hand, during anodic potential scan the film turned transparent, which indicated WO_3_ oxidation followed by de-intercalation of lithium ions.

**Figure 5:**
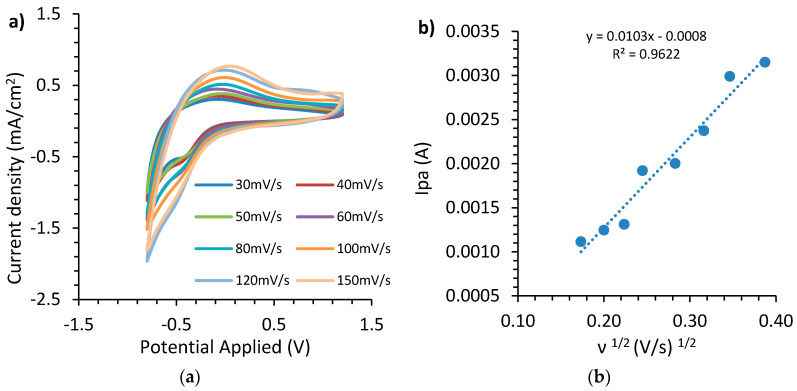
(**a**) WO_3_ cyclic voltammetry curves at various scan rates, (**b**) plot of anodic peak current versus the square root of the scan rate.

An important parameter for the evaluation of electrochromic films is the diffusion coefficient (D). In order to calculate the diffusion coefficient of tungsten trioxide film, cyclic voltammograms at different scan rates namely: 30, 40, 50, 60, 80, 100, 120, and 150 mV/s were used as shown in Figure 5a. It is obvious that the shape of the curve is not altered with increasing scan rate. By using the current peak during anodic scans (i_pa_), as it arises from cyclic voltammograms, the diffusion coefficient of the electrochromic film can be calculated, applying the Randles Sevcik Equation (3) [44,56]:

(3) $$i_{pa}=2.71\times 10^{5}\times n^{\frac{3}{2}}\times A\times C_{o}\times D^{\frac{1}{2}}\times \nu^{\frac{1}{2}}$$

where n is the number of electrons participating in the electrochemical reaction (n = 1 in redox pair W^5+^/W^6+^), A is the total surface area of the working electrode (in cm^2^), C_0_ is the concentration of active ions in the solution (in mol cm^−3^) and ν is the scan rate (in V s^−1^).

Figure 5b indicates a linear relation between current anodic peaks during different scan rates with the square root of the scan rate, which confirms a controlled diffusion behavior. From the slope of the line in Figure 5b and after using Equation (3), the diffusion coefficient for WO_3_ film was calculated to be 0.95 × 10^−16^ cm^2^ s^−1^ for a film with active surface area of 3.89 cm^2^ and 1 M concentration C_0_ of lithium ions in the solution.

**Figure 6:**
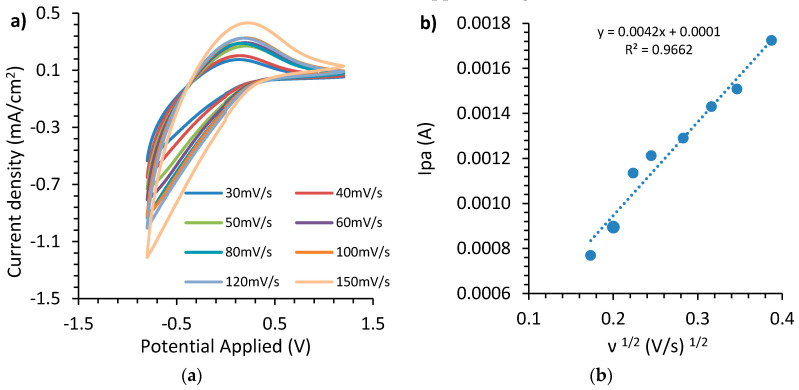
(**a**) Cerium-modified TiO_2_ voltammetry curves at various scan rates, (**b**) plot of anodic peak current versus the square root of the scan rate.

A similar procedure was followed in the case of a cerium-modified TiO_2_ electrode for the evaluation of the diffusion coefficient (D); the results appear in Figure 6a.

Using again Equation (3) and calculating the slope of linear behavior between current anodic peaks during different scan rates with the square root of the scan rate, the diffusion coefficient for the cerium-modified TiO_2_ electrode (Figure 6b) was calculated as 12.87 × 10^−18^ cm^2^ s^−1^.

**Figure 7:**
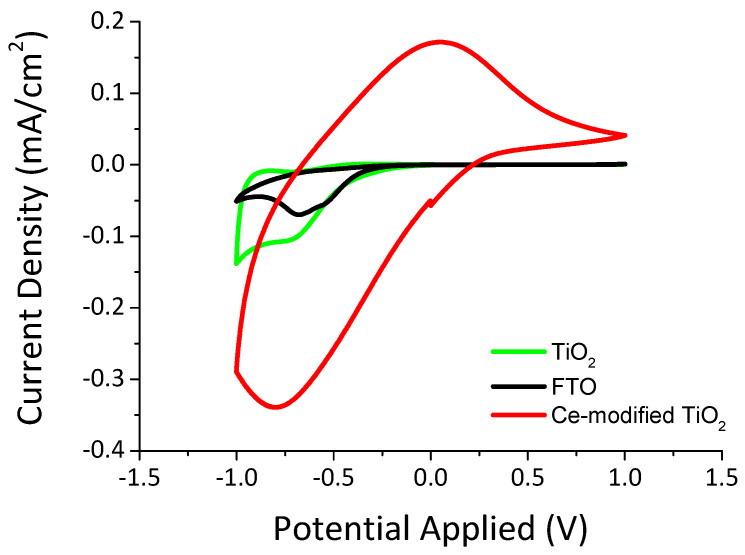
Cyclic voltammograms for SnO_2_:F (FTO), TiO_2_ and Ce-modified TiO_2_ coated glasses measured at a scan rate 10 mV/s.

Figure 7 shows the cyclic voltammograms for the Ce-modified TiO_2_ film on FTO glass in comparison to TiO_2_ on FTO glass and to plain FTO coated glass. The cyclic voltammetry measurements were recorded at a scan rate of 10 mV/s between −1.0 V and 1.0 V. Charge capacity of inserted ions can be obtained from cyclic voltammetry data. Therefore, it was calculated using the following Equation [57]:

(4) $$C=\int \frac{j\times dV}{s}$$

where j corresponds to the current density (mA cm^−2^), V to the voltage applied (V), s to the scan rate (V s^−1^) and C to the charge capacity (mC cm^−2^).

Table 1 shows the charge capacity of inserted ions calculated from Equation (4). It is obvious that the charge capacity of FTO coated glass (4.26 mC cm^−2^) and of TiO_2_ (6.21 mC cm^−2^) is much smaller than the charge capacity calculated for Ce-modified TiO_2_ film (26.77 mC cm^−2^).

In conclusion, Ce-modified TiO_2_ film is appropriate to be used as an ion storage layer at electrochromic devices due to its high charge capacity.

### 3.4. Evaluation of Electrochromic Device

**Figure 8:**
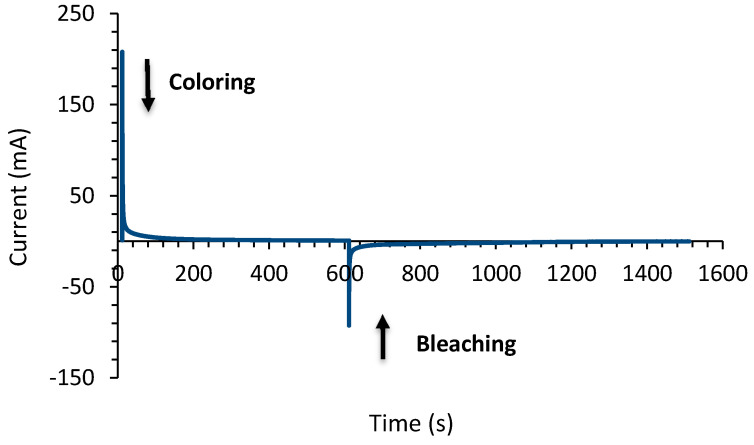
Chronoamperometry curve of the electrochromic cell appearing both at the bleached and the tinted states.

**Figure 9:**
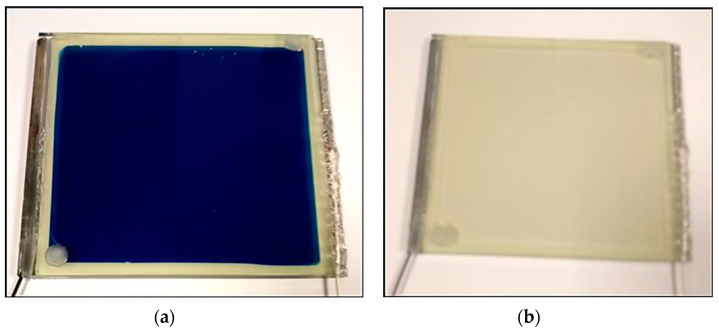
(**a**) Tinted and (**b**) bleached state of the fabricated electrochromic device with dimensions: 9 cm × 10 cm.

For the assessment of the electrochromic device with configuration of Glass/FTO/WO_3_/Quasi-solid-state electrolyte/Ce-modified TiO_2_/FTO/Glass chronoamperometry was used. Chronoamperometry measurements were recorded with the potential being stepped from 0 to +2.5 V for 10 min while performing the coloring procedure and from 0 to −0.3 V for 15 min while performing the bleaching procedure as shown in Figure 8. The fabricated device appeared dark blue in the colored state as depicted in Figure 9a and became transparent after the bleaching cycle as shown at Figure 9b.

**Figure 10:**
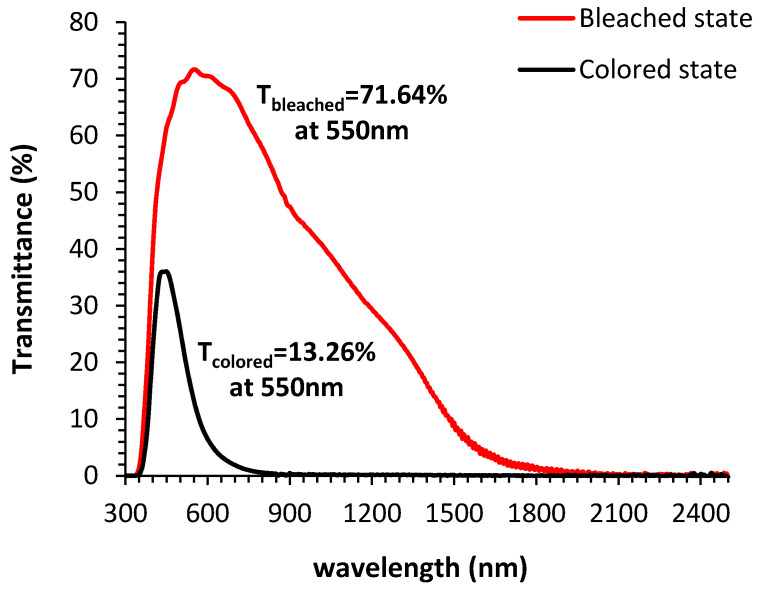
Transmittance of fabricated electrochromic cell at the colored state and the bleached state.

Furthermore, Figure 10 illustrates the corresponding transmittance spectra of the cell at the colored and the bleached states. As can be seen in the figure, the transmittance of the device in the colored state was measured to be 13.26% at 550 nm while at the bleached state it was 71.64% measured at the same wavelength. We conclude that there is an 81.5% reduction of transmittance because of the coloring procedure. Furthermore, we note the obvious reduction of the transmittance at both stages in the infrared region even at the bleached stage (cf. Figure S2). This means that present electrochromic devices, with the use of specific quasi-solid-state electrolyte, could contribute to the thermal relief of a building by substituting the normal glass panes with a specific electrochromic material, due to the obvious reduction of the infrared (thermal) radiation.

**Figure 11:**
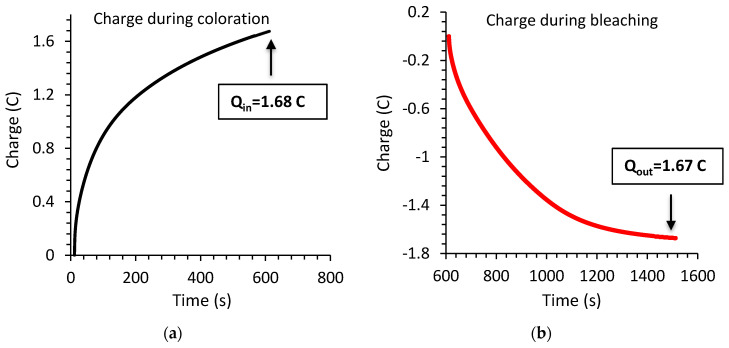
Variation of charge **(a)** inserted into the electrochromic device and **(b)** extracted from the electrochromic device.

Figure 11 represents the variation of charge inserted to and extracted from the electrochromic device during the coloration and bleaching process respectively.

As can be found the device shows an excellent reversibility as the charge inserted into the device (1.68 C) during coloration is equivalent to the charge extracted (1.67 C) from the device during bleaching. A very important parameter in order to evaluate an electrochromic device is coloration efficiency (CE). The CE at a particular wavelength, η(λ), is calculated by the following Equation [58]:

(5) η(λ)=ΔOD(λ)Qin=ΔOD(λ)qinA

therein, Q_in_ is the intercalated charge per unit electrode area (C cm^−2^), q_in_ is the intercalated charge (C) and A is the area of the electrode (cm^−2^). Furthermore ΔOD(λ) is the change in optical density, a dimensionless quantity given as follows [58]:

(6) $$\Delta OD\left(\lambda \right)=\log\frac{T_{b}\left(\lambda \right)}{T_{c}\left(\lambda \right)}$$

where T_b_(λ) and T_c_(λ) is the transmittance of the device in its bleached and tinted state at a particular wavelength (550nm), respectively. Based on Equation (6) the change in optical density was found to be ΔOD(λ) = 0.73. Furthermore, from Equation (5) the coloration efficiency of the electrochromic device is CE = 30.97 cm^2^/C for q_in_ = 1.68 C and A = 71.28 cm^2^ as shown in Table 2.

The long-term cycling stability of the electrochromic cell was also evaluated by observing the loss of optical contrast upon repetitive cycling. The device succeeded to keep more than 93% of its maximum optical contrast during the application of boundary potentials in the form of square-wave pulses for over one hundred and fifty successive cycles with no obvious malfunction (cf. Figure S3).

Finally, in terms of electrochemical energy storage performance, we calculated the energy density (E in Wh cm^−2^), the energy power density (P in W m^−2^) and the areal capacitance (C_a_ in F cm^−2^) of the electrochromic device prepared in this work. The aforementioned calculations were estimated with the use of the following Equations [59]:

(7) $$E=\frac{E_{t}}{A}$$

(8) $$P=\frac{E}{\Delta t}$$

(9) $$C_{a}=\frac{C_{\mathrm{eff}}}{A}$$

where E_t_ (Wh) is the amount of total energy that is released from the device during the discharging procedure, A is the area of the electrode (cm^2^) and Δt is the discharging time (s). Furthermore, C_eff_ (F) is the effective capacitance, which is calculated as follows:

(10) $$E_{t}=\frac{1}{2}C_{eff}V^{2}\Leftrightarrow C_{eff}=\frac{2\times E_{t}}{V^{2}}$$

where V is the voltage of the cell.

As shown in Table 3, the electrochromic device studied in this work has an energy density of 1.95 × 10^−3^ mWh cm^−2^ and a high areal capacitance of 156.2 mF cm^−2^, so it could be a promising candidate for future applications into smart buildings.

## 4. Conclusions

In the above experiments, we presented the good matching between WO_3_ and Ce modified TiO_2_ electrodes in electrochemical and electrochromic properties in large-scale devices using quasi-solid electrolyte. In particular, nanocrystalline WO_3_ and Ce modified TiO_2_ electrodes prepared via the sol-gel method exhibited porous structure consisting of 20–40 and 7–8 nm particle size, respectively. Especially for the counter electrode a very high particle specific area of 151 m^2^/g was measured for Ce modified TiO_2_, which is 25% higher than pristine TiO_2;_ additionally Ce modified TiO_2_ is 26% more porous, which is very important for electrochromic applications. The charge capacity of the Ce-TiO_2_ electrode was 26.77 mC/cm^2^, much higher than 6.21 mC/cm^2^ measured for TiO_2_. The quasi-solid-state electrochromic device in dimensions 9 x 10 cm exhibited a wide optical contrast of 13 to 72% in a cell’s transmittance measured at 550 nm in a low activation voltage window of +2.5 to −0.3 volts. The additional benefit of the energy storage by monitoring high areal capacitance (156.2 mF/cm^2^) makes this electrode combination attractive to smart energy saving applications.

## Figures and Tables

**Table 1:** Comparison of charge capacity of the measured films.

Counter Electrode	Charge Capacity (mC cm^−2^)
SnO_2_:F (FTO)	4.26
TiO_2_/FTO	6.21
Ce-modified TiO_2_/FTO	26.77

**Table 2:** Electrochromic properties of the fabricated device.

EC Device	T_b_ (%) *	T_c_ (%) *	ΔOD(λ)	q_in_ (C)	A (cm^2^)	η (cm^2^/C)
9 cm × 10 cm	71.64	13.26	0.73	1.68	71.28	30.97

^*^ measured at 550 nm.

**Table 3:** Calculation of energy density, energy power density and areal capacitance of the fabricated electrochromic device.

EC Device	E (mWh cm^−2^)	P (mW cm^−2^)	C_a_ (mF cm^−2^)
9 cm × 10 cm	1.95 × 10^−3^	7.82 × 10^−3^	156.2

## Supplementary Materials

The following are available online at https://www.mdpi.com/1996-1944/13/14/3241/s1, Figure S1: XRD of pristine TiO_2_ and cerium modified films, Figure S2: Transmittance (%) vs wavelength for (a) plain glass, (b) FTO glass, (c) tungsten trioxide over FTO glass and (d) complete EC device, Figure S3: Transmittance(%) measured at 550nm vs number of cycles for the 9 cm × 10 cm device.
