# Supplementary material for: Quasi-Solid-State Electrochromic Cells with Energy Storage Properties Made with Inkjet Printing
Source: Materials (Basel). 2020 Jul 21;13(14):3241. doi: 10.3390/ma13143241 (PMC7412062; doi:10.3390/ma13143241)
Supplement: Supplementary file 1 [file materials-13-03241-s001.pdf]

materials

Supplementary Materials

Quasi-Solid-State Electrochromic Cells with Energy Storage Properties Made with Inkjet Printing

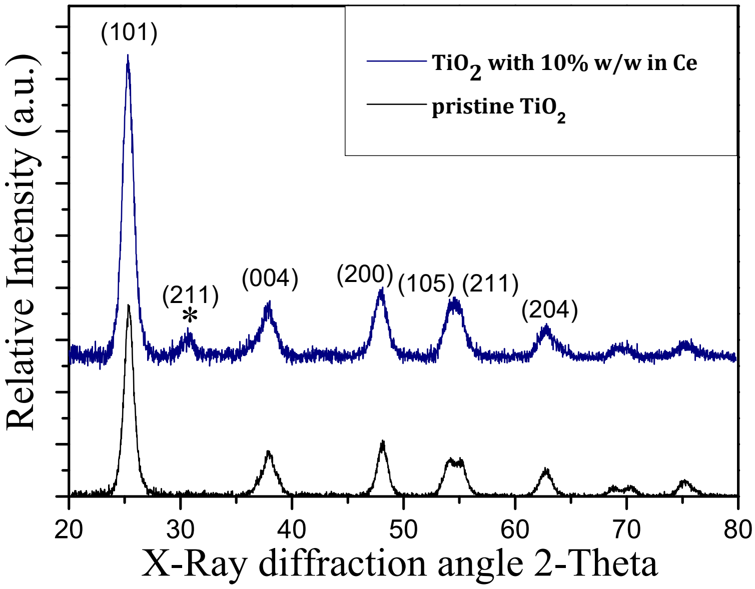

Figure S1. XRD of pristine TiO_2 and cerium modified films.

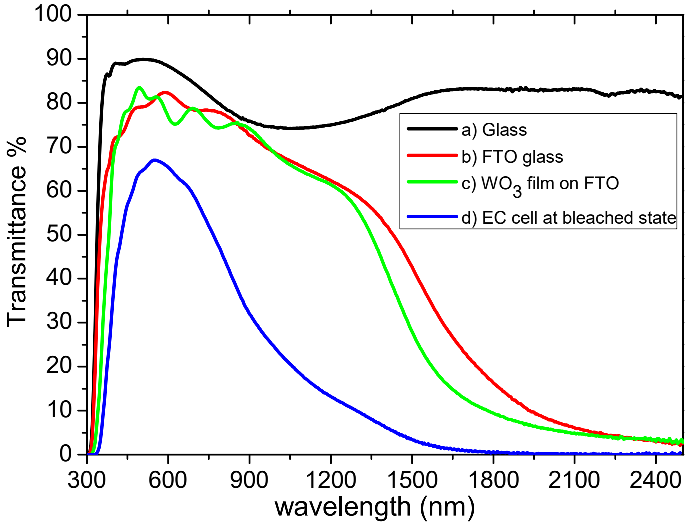

Figure S2. Transmittance (%) vs wavelength for (**a**) plain glass, (**b**) FTO glass, (**c**) tungsten trioxide over FTO glass and (**d**) complete EC device.

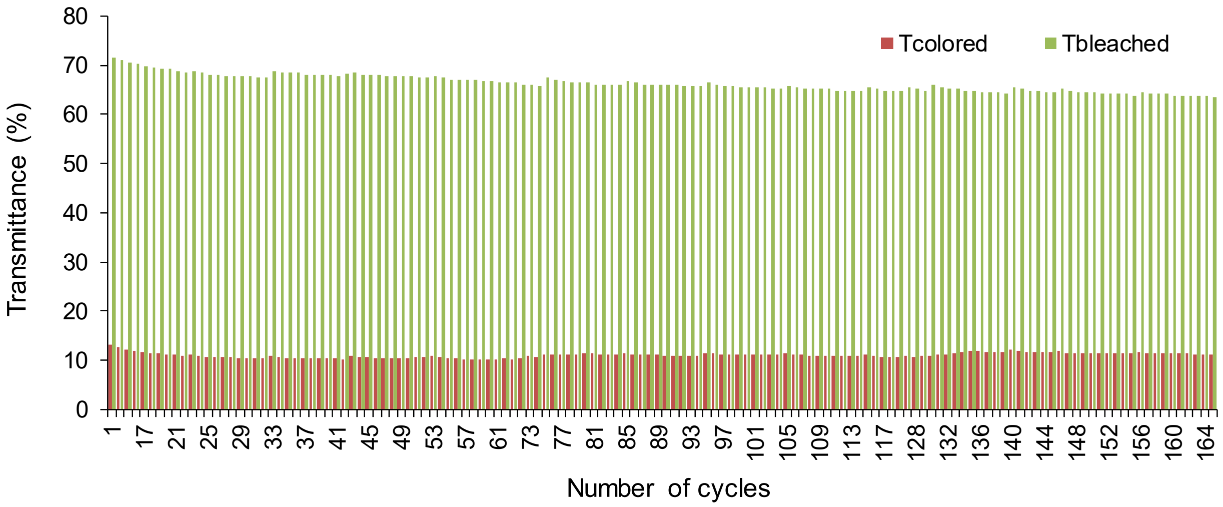

Figure S3. Transmittance(%) measured at 550 nm vs number of cycles for the 9 cm × 10 cm device.

© 2020 by the authors. Submitted for possible open access publication under the terms and conditions of the Creative Commons Attribution (CC BY) license (http://creativecommons.org/licenses/by/4.0/).
